# Effect of the chronic medication use on outcome measures of hospitalized COVID-19 patients: Evidence from big data

**DOI:** 10.3389/fpubh.2023.1061307

**Published:** 2023-02-24

**Authors:** Mohammad-Reza Malekpour, Mohsen Abbasi-Kangevari, Ali Shojaee, Sahar Saeedi Moghaddam, Seyyed-Hadi Ghamari, Mohammad-Mahdi Rashidi, Alireza Namazi Shabestari, Mohammad Effatpanah, Mohammadmehdi Nasehi, Mehdi Rezaei, Farshad Farzadfar

**Affiliations:** ^1^Non-Communicable Diseases Research Center, Endocrinology and Metabolism Population Sciences Institute, Tehran University of Medical Sciences, Tehran, Iran; ^2^Department of Health Management and Economics, School of Public Health, Tehran University of Medical Sciences, Tehran, Iran; ^3^Kiel Institute for the World Economy, Kiel, Germany; ^4^Department of Geriatric Medicine, School of Medicine, Tehran University of Medical Sciences, Tehran, Iran; ^5^National Center for Health Insurance Research, Iran Health Insurance Organization, Tehran, Iran; ^6^Department of Pediatrics, Imam Khomeini Hospital Complex, School of Medicine, Tehran University of Medical Sciences, Tehran, Iran; ^7^Pediatric Neurology Department, Mofid Children Hospital, Shahid Beheshti University of Medical Sciences, Tehran, Iran; ^8^Department of Orthopedics, School of Medicine, Tehran University of Medical Sciences, Tehran, Iran; ^9^Endocrinology and Metabolism Research Center, Endocrinology and Metabolism Clinical Sciences Institute, Tehran University of Medical Sciences, Tehran, Iran

**Keywords:** COVID-19, non-communicable diseases, big data, frequent pattern mining, Anatomical Therapeutic Chemical, pandemic

## Abstract

**Background:**

Concerns about the role of chronically used medications in the clinical outcomes of the coronavirus disease 2019 (COVID-19) have remarkable potential for the breakdown of non-communicable diseases (NCDs) management by imposing ambivalence toward medication continuation. This study aimed to investigate the association of single or combinations of chronically used medications in NCDs with clinical outcomes of COVID-19.

**Methods:**

This retrospective study was conducted on the intersection of two databases, the Iranian COVID-19 registry and Iran Health Insurance Organization. The primary outcome was death due to COVID-19 hospitalization, and secondary outcomes included length of hospital stay, Intensive Care Unit (ICU) admission, and ventilation therapy. The Anatomical Therapeutic Chemical (ATC) classification system was used for medication grouping. The frequent pattern growth algorithm was utilized to investigate the effect of medication combinations on COVID-19 outcomes.

**Findings:**

Aspirin with chronic use in 10.8% of hospitalized COVID-19 patients was the most frequently used medication, followed by Atorvastatin (9.2%) and Losartan (8.0%). Adrenergics in combination with corticosteroids inhalants (ACIs) with an odds ratio (OR) of 0.79 (95% confidence interval: 0.68–0.92) were the most associated medications with less chance of ventilation therapy. Oxicams had the least OR of 0.80 (0.73–0.87) for COVID-19 death, followed by ACIs [0.85 (0.77–0.95)] and Biguanides [0.86 (0.82–0.91)].

**Conclusion:**

The chronic use of most frequently used medications for NCDs management was not associated with poor COVID-19 outcomes. Thus, when indicated, physicians need to discourage patients with NCDs from discontinuing their medications for fear of possible adverse effects on COVID-19 prognosis.

## 1. Introduction

Non-communicable diseases (NCDs), accounting for about three-quarters of global deaths and one-third of disability-adjusted life years (DALYs) in 2019, place the most significant burden of disease on public health (1). However, due to their unknown nature and speed of spread, emerging infectious diseases can pose tremendous challenges to the health system. The coronavirus disease 2019 (COVID-19), the latest and most widespread pandemic in the twenty-first century, has resulted in more than 600 million cases and nearly 6.4 million deaths until September 2022 (2).

Several studies have shown the interaction between NCDs and COVID-19 (3–5). One of the most controversial topics with contradictory results is the effect of chronic use of medications on the clinical outcomes of COVID-19. For instance, some research suggested that angiotensin-converting enzyme (ACE) inhibitors have been associated with an increased risk of death from COVID-19, while others indicated an association between desirable clinical outcomes and the chronic use of ACE inhibitors (6, 7).

According to a World Health Organization (WHO) survey of 155 countries, 53%, 49%, and 45% of the countries had partially disrupted services for hypertension, diabetes, and cancer treatment, respectively, during the COVID-19 pandemic (8). Causes such as quarantine, self-isolation, and unprecedented load on the health systems, have been shown to have a detrimental effect on the quality of NCDs management, especially in the early days of the COVID-19 pandemic (9–12). However, uncertainty about the role of chronic medication use in the clinical outcome of COVID-19 has an abiding potential for the breakdown of NCDs management by imposing ambivalence toward medication continuation.

Despite the interest in the effect of individual medications with chronic use on COVID-19 clinical outcomes, the role of medication combinations has not been explored in depth. This study aimed to investigate whether single or combinations of widely used medications in treating chronic diseases was associated with clinical outcomes of COVID-19.

## 2. Materials and methods

### 2.1. Design and data acquisition

This retrospective cohort study was conducted on hospitalized COVID-19 patients in Iran. Data of hospitalized COVID-19 patients were obtained from the Iranian COVID-19 registry, which included about 1.2 million patients from February 1st, 2020, to June 8th, 2021. The diagnosis of COVID-19 was made based on the results of Reverse Transcription Polymerase Chain Reactions (RT-PCR) for SARS-CoV-2, or lung Computed Tomography (CT) scan. All patients received supportive and antiviral treatment according to the national guidelines. Extracted variables from the Iranian COVID-19 registry consisted of age, sex, admission date, chronic comorbidities, length of hospital stay, Intensive Care Unit (ICU) admission, ventilation therapy, and death. Cardiovascular diseases (CVDs), chronic respiratory diseases (CRDs), diabetes mellitus (DM), and malignancies were inspected as chronic comorbidities. Claimed prescriptions data were retrieved from Iran Health Insurance Organization (IHIO), including medication names and quantities of prescriptions for the years 2018 and 2019. To investigate the burden on both COVID-19 patients and the healthcare system, outcome measures with the most prominent health and economic impacts were included in this study. The primary outcome of this study was death, and secondary outcomes included length of hospital stay, ICU admission, and ventilation therapy.

### 2.2. Data preprocessing

**Figure 1:**
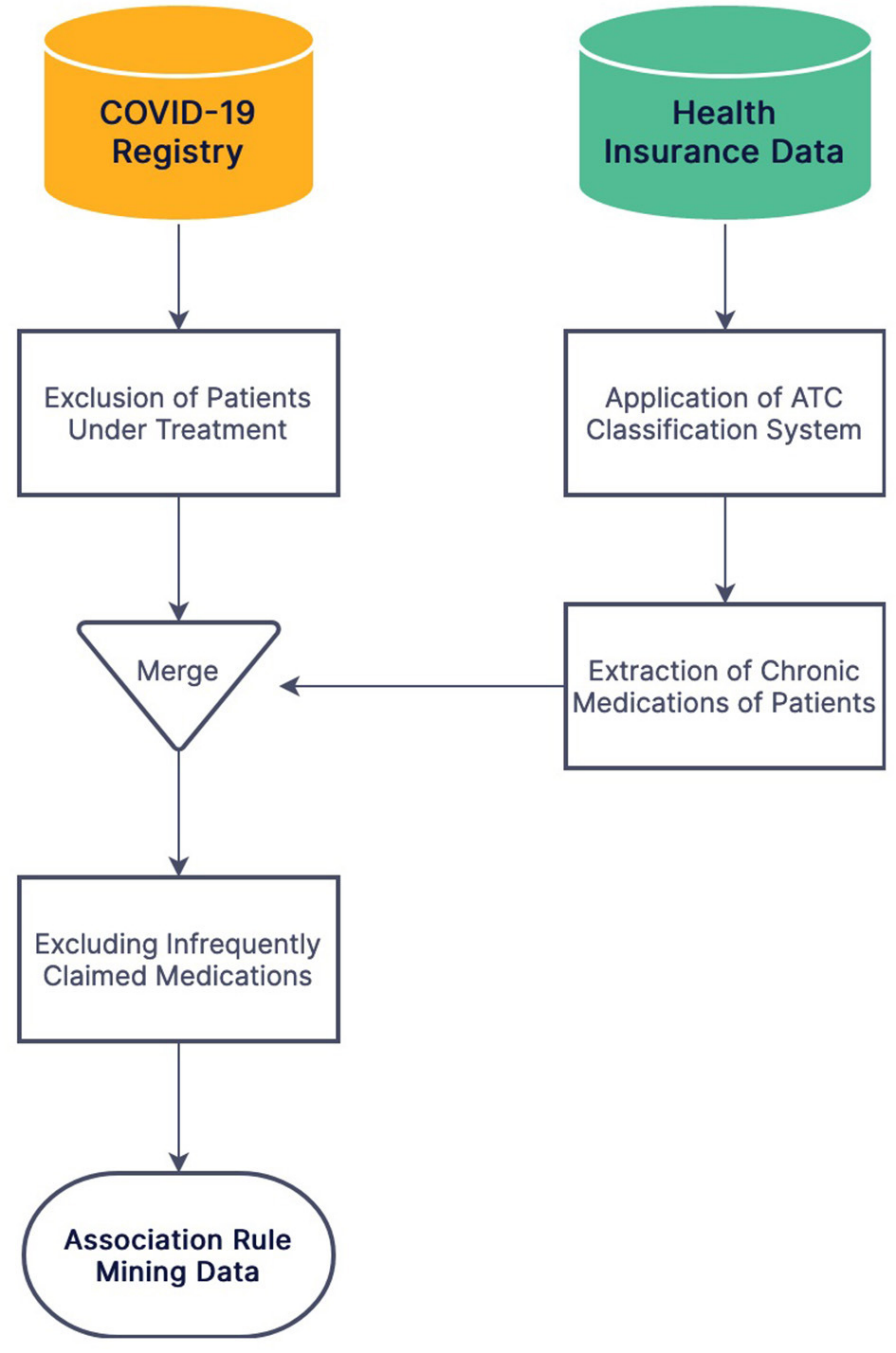
The flowchart of data preprocessing and merging stages of the Iranian COVID-19 registry and IHIO.

The national identification number was used for the record linkage of the two databases. COVID-19 patients under treatment were excluded from the study due to an unknown outcome. Admission dates were divided into 3-month intervals to be included in the statistical models due to the different virulence of SARS-CoV-2 variants and vital equipment shortage during the pandemic surges. The Anatomical Therapeutic Chemical (ATC) classification system was applied to medication names for obtaining standard coding. Among five levels of granularity in the ATC classification system, level four was used for medication grouping (ATC groups) in this study. The level five ATC codes claimed at least four times during the 2018–2019 period were considered chronically used medications for each patient. Chronically used medications claimed by less than one percent of COVID-19 patients were excluded from the investigation (Figure 1).

### 2.3. Association rule mining

To investigate the effect of medication combinations on COVID-19 outcomes, the frequent pattern growth algorithm, an efficient association rule mining algorithm, was utilized for item set extraction (13). For elimination of uncommon combinations, the support hyperparameter was used, defined as below:


$$\begin{array}{l} Support_{\left(A,B\right)}=\frac{Number\;of\;Patients\;with\;Chronic\;Use\;of\;A\;and\;B}{Total\;Number\;of\;Patients} \end{array}$$

Among all combinations, two- and three-medication sets with support of at least 0.01 were included in the study.

### 2.4. Statistical analysis

The accelerated failure time (AFT) model was utilized with the Weibull survival function to assess medications' association with length of hospital stay. The logistic regression model was used to evaluate the association of medications with binary outcomes, including ICU admission, ventilation therapy, and death. The models were adjusted for age, gender, admission date, chronic comorbidities, and receiving ventilation therapy. Moreover, the use of each drug of the set alone was also considered a covariate. The 0.05 alpha level was used for significance inference and confidence interval (CI) computation of coefficients and odds ratio (OR) in all statistical analyses. Apache Spark, version 3.2.0, a unified big data processing engine, was utilized for data preprocessing and association rule mining (14). Statistical analyses were performed using Python libraries Statsmodels, version 0.13, and Lifelines, version 0.27 (15, 16).

### 2.5. Ethical considerations

This study was conducted according to the guidelines of the Declaration of Helsinki, and approved by Research Ethics Committees of Endocrine and Metabolism Research Institute, Tehran University of Medical Sciences (IR.TUMS.EMRI.REC.1400.046).

## 3. Results

### 3.1. Overview

A total of 258,917 patients were identified by joining the IHIO database and the Iranian COVID-19 registry following the exclusion of patients under treatment. Overall, 53.0% of patients were female, and the median age was 63 [interquartile range (IQR): 48–75] years. The last and first quarters of 2020 had the highest and lowest number of COVID-19 patients, respectively (Table 1). CVDs with affecting 30.3% of patients, were the leading chronic comorbidities, followed by DM (19.7%) and CRDs (5.6%). The median length of hospital stay was 5 days (IQR: 3–8) and 7 days (IQR: 3–13) in recovered and deceased patients, respectively. Among the study population, 21,363 (8.3%) were admitted to ICU, 18,564 (7.2%) underwent ventilation therapy, and 41,618 (16.1%) were deceased due to COVID-19.

**Table 1:** Distribution of admission dates, chronic comorbidities, and outcomes among hospitalized COVID-19 patients.

	**Patients count (percent)**
**Admission date**	
2020Q4	71,494 (27.6%)
2020Q3	51,456 (19.9%)
2021Q2	42,582 (16.4%)
2020Q2	39,369 (15.2%)
2021Q1	37,440 (14.5%)
2020Q1	16,576 (6.4%)
**Chronic comorbidity**	
CVDs	78,446 (30.3%)
DM	50,917 (19.7%)
CRDs	14,628 (5.6%)
DM (insulin therapy)	11,441 (4.4%)
Malignancies	4,924 (1.9%)
**Outcomes**	
Death	41,618 (16.1%)
ICU admission	21,363 (8.3%)
Ventilation therapy	18,564 (7.2%)

### 3.2. Drug utilization

**Figure 2:**
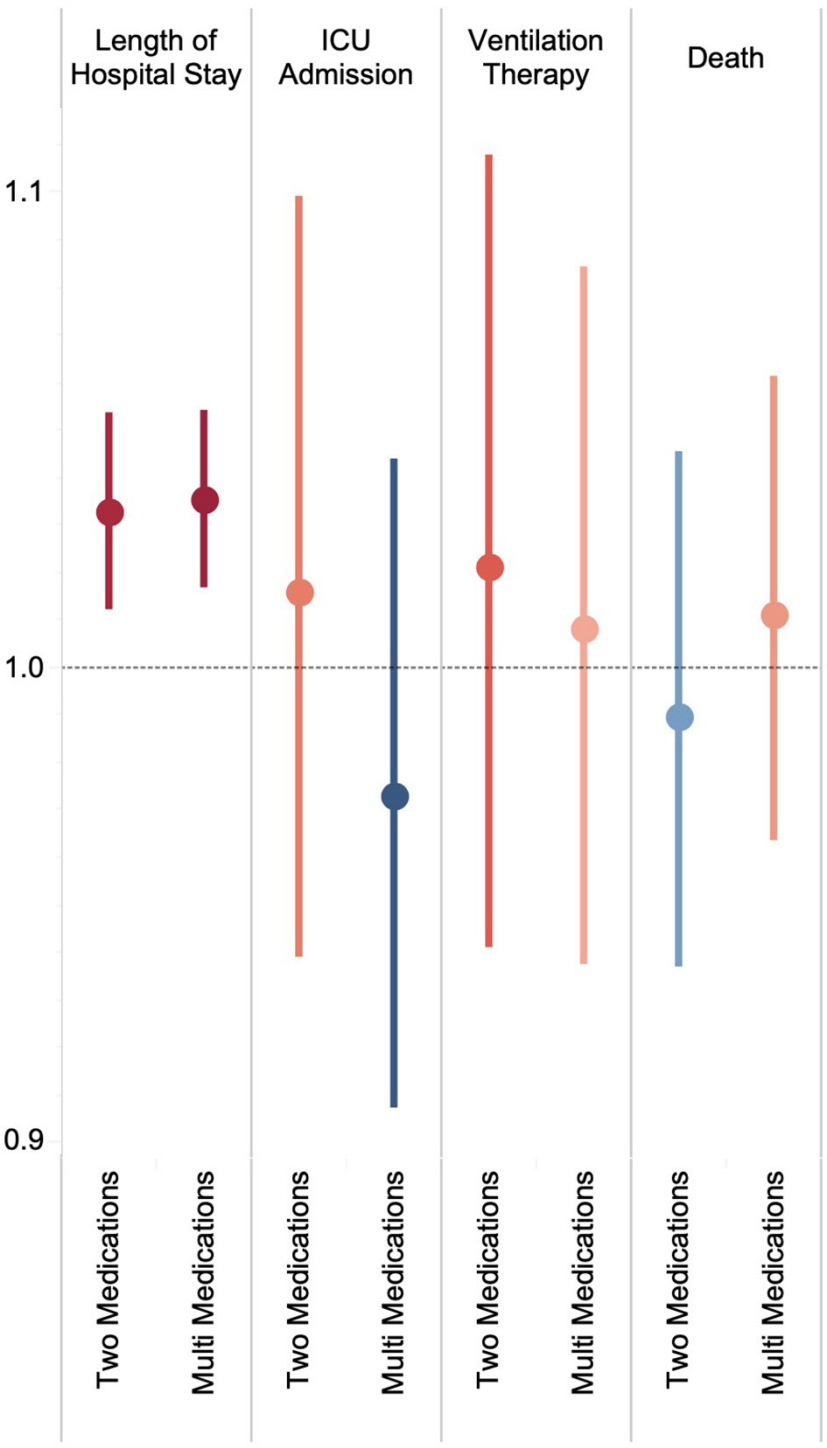
Change in length of hospital stay and ORs of ICU admission, ventilation therapy, and death by the number of chronically used medications.

Of the total 607 chronically used medications, 42 ATC groups and 42 level five ATC codes were claimed by at least 1% of the study population. Platelet aggregation inhibitors excluding Heparin (B01AC), plain Angiotensin II receptor blockers (ARBs; C09CA), and HMG CoA reductase inhibitors (C10AA) were the most frequent ATC groups with chronic use in 10.8%, 9.4%, and 9.4% of patients, respectively (Supplementary Data Sheet 1). Besides, Aspirin (10.8%), Atorvastatin (9.2%), and Losartan (8.0%) were the leading medications in chronic use (Supplementary Data Sheet 2). Aspirin-Atorvastatin (5.7%) and Aspirin-Atorvastatin-Losartan (2.5%) had the highest frequency of chronic use among two- and three-medication sets, respectively (Supplementary Data Sheet 3). There was no significant difference between chronic use of a single medication vs. two or more than two medications in ICU admission, need for ventilation therapy, and death (Figure 2).

### 3.3. Outcome measures

#### 3.3.1. Medication groups

**Figure 3:**
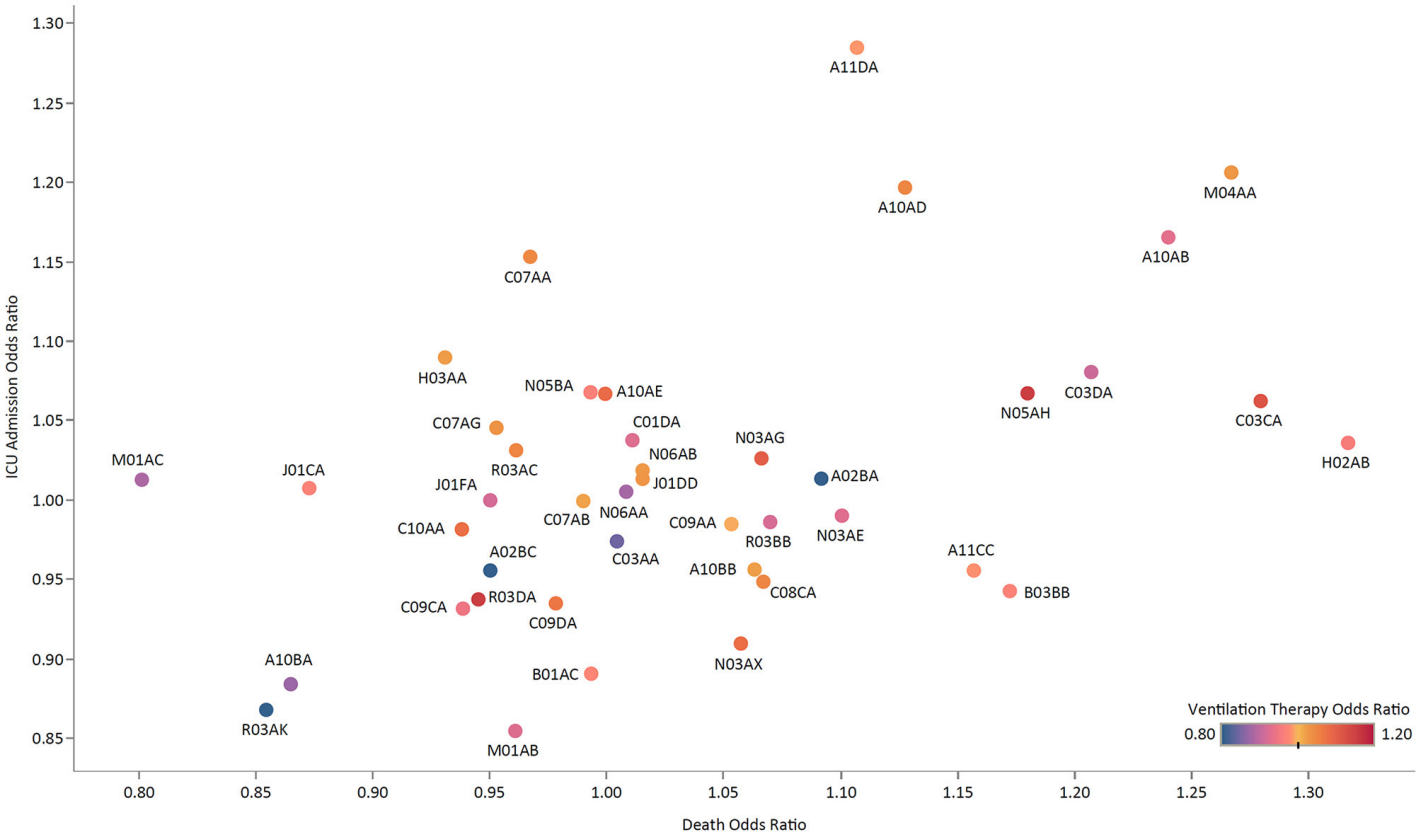
The ORs of ICU admission vs. death for medication groups, colored by the OR of ventilation therapy.

The most associated ATC groups with the prolongation of hospital stay were preparations inhibiting uric acid production (M04AA) and fatty acid derivatives antiepileptics (N03AG) with 11.6% (95% CI: 7.2–16.1) and 11.3% (7.7–15.0) increase, respectively (Supplementary Table 1). Among ATC groups, chronic use of acetic acid derivatives and related substances (M01AB) with an OR of 0.85 (0.76–0.96) had the least association with ICU admission (Figure 3; Supplementary Table 1). Adrenergics in combination with corticosteroids inhalants (ACIs) had the lowest OR for undergoing ventilation therapy [0.79 (0.68–0.92)], followed by plain Thiazides [C03AA; 0.84 (0.72–0.98)] and Biguanides [A10BA; 0.87 (0.80–0.94)]. Regarding the association of chronic use of ATC groups with death, Oxicams (M01AC) had a significantly low OR of 0.80 (0.73–0.87), followed by ACIs [R03AK; 0.85 (0.77–0.95)] and Biguanides [A10BA; 0.86 (0.82–0.91)].

#### 3.3.2. Single medications

**Figure 4:**
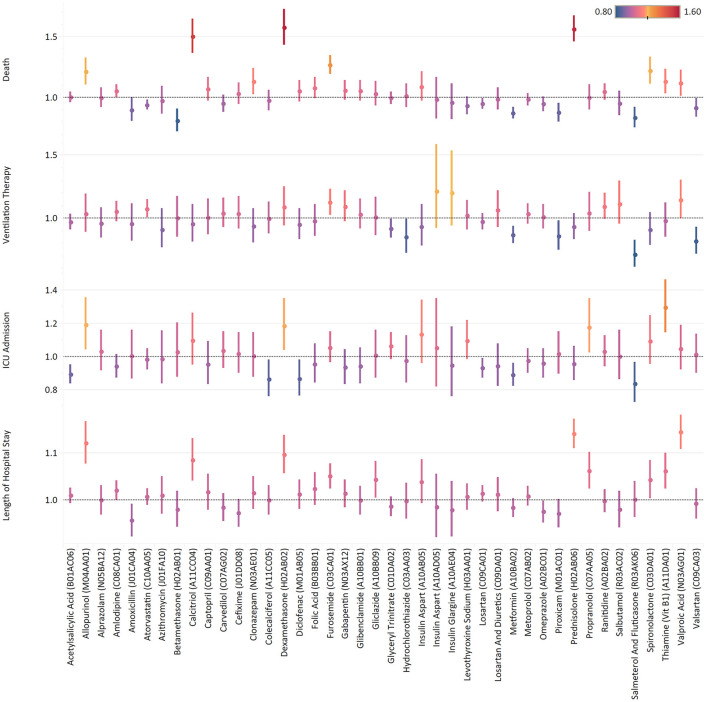
Change in length of hospital stay along with ORs of ICU admission, ventilation therapy, and death by medications.

Valproic acid with a 14.4% (10.8–18.1) increase in hospital stay, along with Prednisolone [14.2% (11.2–17.3)] and Allopurinol [12.1% (7.6–16.6)] were the most associated medications with the lengthening of hospital stay (Figure 4; Supplementary Table 2). Salmeterol/Fluticasone inhaler with an OR of 0.84 (0.73–0.97) was the leading medication in negative association with ICU admission, followed by Cholecalciferol [0.84 (0.72–0.96)], and Diclofenac [0.87 (0.76–0.98)]. Regarding the need for ventilation therapy, Salmeterol/Fluticasone inhaler [OR: 0.71 (0.61–0.83)], Valsartan [OR: 0.82 (0.72–0.93)], and Hydrochlorothiazide [OR: 0.85 (0.73–0.99)] had the lowest association. Similarly, Salmeterol/Fluticasone inhaler [OR: 0.84 (0.76–0.92)] and Metformin [OR: 0.87 (0.83–0.92)], respectively, were the second and third least associated medications with COVID-19 death. While chronic use of Betamethasone was negatively associated with death [OR: 0.81 (0.72–0.91)] more than any other medication, Dexamethasone [OR: 1.58 (1.44–1.73)] was among the leading medications in positive association with death.

#### 3.3.3. Two-medication sets

The most associated two-medication sets with the decrease in hospital stay were Amlodipine-Hydrochlorothiazide [16.4% (9.3–22.8)], Carvedilol-Spironolactone [15.7% (8.7–22.1)], and Aspirin-Valproic acid [15.3% (8.6–21.4); Supplementary Table 3]. Hydrochlorothiazide-Metformin with an OR of 0.64 (0.46–0.90) had the least association with ICU admission, followed by Salbutamol-Salmeterol/Fluticasone [0.66 (0.47–0.94)], and Alprazolam-Metformin [0.72 (0.52–0.98)]. In contrast, Folic acid-Losartan and Losartan-Spironolactone were the most related two-medication sets to ICU admission, with ORs of 1.35 (1.05–1.74) and 1.30 (1.01–1.68), respectively. While Glibenclamide-Losartan with an OR of 0.79 (0.63–0.99) was the least associated two-medication set with the need for ventilation therapy, Gliclazide-Losartan [1.63 (1.22–2.17)] was the most associated set. Among all two-medication sets, Folic acid-Prednisolone with a significant OR of 0.70 (0.59–0.84) had the least association with death, followed by Carvedilol-Spironolactone [0.73 (0.61–0.88)].

#### 3.3.4. Three-medication sets

Aspirin-Carvedilol-Spironolactone with a 17.8% (10.7–24.4) decrease in length of hospital stay was the most negatively associated three-medication set, followed by Aspirin-Glyceryl Trinitrate-Spironolactone [16.8% (9.9–23.2); Supplementary Table 4]. No three-medication set was significantly associated with reducing ICU admission or the need for ventilation therapy. The least associated three-medication sets with death were Aspirin-Furosemide-Spironolactone and Amlodipine-Atorvastatin-Metformin, with ORs of 0.72 (0.61–0.85) and 0.73 (0.63–0.84), respectively.

## 4. Discussion

Aspirin, Atorvastatin, and Losartan were the leading chronically used medications among patients hospitalized due to COVID-19. ACIs were the most associated medications with less chance of ventilation therapy, followed by plain Thiazides and Biguanides. Oxicams had the most inverse association with death, followed by ACIs and Biguanides.

Hypertension is associated with severe COVID-19 (17, 18). There has been a growing body of literature investigating the potential role of various antihypertensive agents in COVID-19 prognosis. The results of this study suggested that Amlodipine-Hydrochlorothiazide was associated with a 16% decrease in hospital stay. Among ARBs, Valsartan was associated with 18% less likelihood of ventilation therapy. Moreover, the chronic use of plain Thiazides was associated with a 15% lower risk of ventilation therapy during COVID-19 hospitalization. There is evidence that ACE inhibitors/ARBs use was associated with a significantly decreased risk of COVID-19 mortality (19). Moreover, they were not associated with significantly increased chances of receiving ICU care (20). They may even have superior beneficial effects on treating hypertension during the COVID-19 pandemic (21). Thus, continuing ACE inhibitors/ARBs during the COVID-19 pandemic can be a suitable strategy (22), as current guidance suggests (23).

The chronic use of Biguanides was associated with about 13% lower mortality due to COVID-19. Similarly, a meta-analysis reported that Metformin was associated with 34% (22–44) lower COVID-19 mortality (24). While the chronic use of Metformin was associated with a 13% lower probability of receiving ventilation therapy, a meta-analysis did not identify a statistically significant association between Metformin and intubation (24). The underlying cause of the association between chronic Metformin use and death due to COVID-19 is yet to be understood. Nevertheless, there is evidence that Metformin could alter the expression and stability of ACE-2, a target of SARS-CoV-2 (25, 26), while also reducing tumor necrosis factor alpha (TNF-α) (27, 28), a proinflammatory cytokine involved in the prognosis of COVID-19 (29).

Due to evidence suggestive of possible links between non-steroidal anti-inflammatory drugs (NSAIDs) and respiratory or cardiovascular adverse effects in several settings, regular NSAIDs use was not recommended as the first-line option for managing the symptoms of COVID-19 (30). However, further evidence suggested that NSAIDs use was not associated with poor COVID-19 outcomes (31). In this study, chronic NSAIDs use was associated with a 14% lower ICU admission due to COVID-19. Notably, chronic Diclofenac users had a 13% lower chance of ICU admission due to COVID-19. Furthermore, Piroxicam was associated with a 14% less risk of ventilation therapy and a 12% less mortality due to COVID-19. Currently, there seems to be no evidence suggesting that clinicians should refrain from or discontinue NSAIDs in patients with COVID-19 upon proper indication (32).

Reports on the impact of inhaled corticosteroids (ICS) on COVID-19 clinical outcomes have been discrepant (33). In this study, Salmeterol/Fluticasone inhaler was associated with 16%, 29%, and 16% reductions in ICU admission, ventilation therapy, and death due to COVID-19, respectively. A study among more than 800,000 patients with asthma reported a non-significant increase in COVID-19-related mortality associated with ICS use (34). While the potential beneficial effects of chronic ICS use, alone or in combination with adrenergics, are yet to be confidently established, there seems to be sufficient evidence for the continuation of these medications when indicated (35).

While the administration of Dexamethasone in hospitalized patients with COVID-19 resulted in lower all-cause mortality (36, 37), the chronic use of Dexamethasone in this study was associated with a 58% higher mortality due to COVID-19. Furthermore, chronic Prednisolone users tended to stay in hospital 14% longer. Similarly, a study reported that the use of systemic glucocorticoids prior to COVID-19 hospitalization was associated with higher mortality (38). Thus, the administration of systemic glucocorticoids needs to be with extra caution during the COVID-19 pandemic. In the meantime, patients who need to take such medications for extended periods must beware of their short-, mid-, and long-term adverse effects, especially during the pandemic. In contrast to Dexamethasone, the chronic use of Betamethasone was associated with a 19% lower mortality due to COVID-19, which needs to be validated and investigated in future studies.

The NCDs pandemic has existed prior to the COVID-19 era, and CVDs, cancers, CRDs, and DM are the four groups of diseases responsible for more than 80% of all NCDs mortality (39, 40). Similarly, in this study, 30% of participants had CVDs, nearly 20% had DM, and some 6% had CRDs. NCDs have resulted in a double burden due to the COVID-19 pandemic, as patients with HTN, DM, CRDs, and CVDs have been reportedly more likely to have poor COVID-19 outcomes (41, 42). COVID-19 pandemic has inevitably resulted in rapid exploitation of healthcare system resources and leaving health systems tremendously overstretched (43). The significant disruptions to conventional NCDs care could lead to increased morbidity and mortality in the long term (44, 45). In the meantime, poor adherence to prescribed medications for NCDs, also fueled by fear of potential adverse effects on COVID-19 infection, could complicate the management of NCDs. In this sense, the present study provides evidence for continuing the most used medications for NCDs when indicated.

### 4.1. Strengths and limitations

This study is the first nationwide study to investigate whether single or combinations of widely used medications in treating chronic diseases affect clinical outcomes of COVID-19, based on the real-world data of more than 250,000 hospitalized patients. The strength of this study lies in a large sample and data gathering since the early days of the outbreak in Iran. Moreover, chronic use of medications was investigated based on the patients' prescriptions from 2 years before the admission date due to COVID-19. Despite the interest in the effect of individual medications with chronic use on COVID-19 clinical outcomes, the role of medication combinations has not been explored in depth.

We also acknowledge the limitations of this study. The focus of this study was the chronic use of medications; hence, the findings have limited implications in the context of the acute use of medications. Moreover, the results of this study do not reflect the potential biochemical pathways through which chronic use of medications may interact with SARS-CoV-2; thus, further *in-vitro*/*in-vivo* studies are required in this sense. Another noteworthy limitation of this study was neglecting the role of disease control due to the lack of an integrated electronic healthcare system. Similarly, the role of CVDs, DM, and CRDs could be adjusted while performing the statistical analysis. The effects of other comorbidities could not be investigated due to the lack of data. Thus, the results of this study need to be interpreted with caution.

### 4.2. Conclusions

The chronic use of most frequently used medications for NCDs management was not associated with poor COVID-19 outcomes. Thus, based on the available evidence and when indicated, physicians need to discourage patients with NCDs, particularly those with CVDs, DM, or CRDs, from discontinuing their medications for fear of possible adverse effects on COVID-19 prognosis. In the meantime, future studies need to investigate the clinical/biochemical role of acute/chronic medication use in the context of COVID-19.

## Data availability statement

The data used in this study is owned by IHIO and Iranian COVID-19 registry. Therefore, authors are not allowed to share the data publicly or privately. However, any researcher with written permission from IHIO and Iranian COVID-19 registry can request to obtain the anonymized data. Requests to access these datasets should be directed to IHIO.gov.ir.

## Ethics statement

This study was conducted according to the guidelines of the Declaration of Helsinki and approved by Research Ethics Committees of Endocrine and Metabolism Research Institute, Tehran University of Medical Sciences (IR.TUMS.EMRI.REC.1400.046).

## Author contributions

Conceptualization: FF and M-RM. Methodology, formal analysis, and visualization: M-RM. Software: SS and M-RM. Validation: MA-K, S-HG, and SS. Investigation and data curation: M-RM, MA-K, S-HG, and M-MR. Resources: AS, AN, ME, MN, and MR. Writing—original draft preparation: M-RM, MA-K, and S-HG. Writing—review and editing: FF, SS, M-RM, and MA-K. Supervision: FF. Project administration: AS. All authors contributed to the article and approved the submitted version.
