# Supplementary material for: Effect of the chronic medication use on outcome measures of hospitalized COVID-19 patients: Evidence from big data
Source: Front Public Health. 2023 Feb 24;11:1061307. doi: 10.3389/fpubh.2023.1061307 (PMC9998941; doi:10.3389/fpubh.2023.1061307)
Supplement: Supplementary file 5 [file Data_Sheet_1.PDF]

Medication Group (ATC)	Total	Recovered	ICU Admission	Ventilation Therapy	Death
Platelet Aggregation Inhibitors Excl. Heparin (B01AC)	10.83%	9.95%	12.70%	12.65%	15.40%
Angiotensin II Receptor Blockers (ARBs), Plain (C09CA)	9.43%	8.74%	11.34%	10.78%	13.03%
HMG CoA Reductase Inhibitors (C10AA)	9.37%	8.73%	11.20%	11.05%	12.73%
Biguanides (A10BA)	6.34%	6.08%	6.97%	6.89%	7.69%
Beta Blocking Agents, Selective (C07AB)	5.44%	5.06%	6.56%	6.28%	7.43%
Glucocorticoids (H02AB)	5.34%	5.06%	5.68%	5.51%	6.81%
Organic Nitrates (C01DA)	5.03%	4.46%	6.51%	5.87%	7.99%
Dihydropyridine Derivatives (C08CA)	4.61%	4.21%	5.57%	5.55%	6.70%
Sulfonamides, Plain (C03CA)	3.45%	2.93%	4.87%	4.51%	6.18%
H2-Receptor Antagonists (A02BA)	3.36%	3.13%	3.86%	3.85%	4.54%
Sulfonylureas (A10BB)	3.01%	2.84%	3.41%	3.49%	3.93%
Proton Pump Inhibitors (A02BC)	2.95%	2.79%	3.22%	3.24%	3.78%
Insulins And Analogues For Injection, Long-Acting (A10AE)	2.74%	2.51%	3.32%	3.08%	3.96%
Vitamin D And Analogues (A11CC)	2.70%	2.47%	2.95%	2.91%	3.89%
Insulins And Analogues For Injection, Fast-Acting (A10AB)	2.70%	2.46%	3.34%	2.87%	3.95%
Benzodiazepine Derivatives (N05BA)	2.56%	2.42%	3.00%	2.70%	3.27%
Acetic Acid Derivatives And Related Substances (M01AB)	2.17%	2.09%	2.09%	2.23%	2.60%
Alpha And Beta Blocking Agents (C07AG)	2.15%	1.93%	2.88%	2.60%	3.29%
Selective Serotonin Reuptake Inhibitors (N06AB)	2.08%	2.01%	2.29%	2.19%	2.43%
Other Antiepileptics (N03AX)	2.05%	1.93%	2.24%	2.38%	2.72%
Thyroid Hormones (H03AA)	1.92%	1.88%	2.23%	2.05%	2.13%
Insulins And Analogues For Injection, Intermediate- Or Long-Acting Combined With Fast-Acting (A10AD)	1.75%	1.59%	2.24%	2.07%	2.62%
Adrenergics In Combination With Corticosteroids Or Other Drugs, Excl. Anticholinergics (R03AK)	1.75%	1.64%	1.93%	1.86%	2.33%
Third-Generation Cephalosporins (J01DD)	1.72%	1.67%	1.83%	1.83%	2.00%
Oxicams (M01AC)	1.71%	1.68%	1.75%	1.65%	1.85%
Folic Acid And Derivatives (B03BB)	1.62%	1.48%	1.78%	1.79%	2.34%
ACE Inhibitors, Plain (C09AA)	1.56%	1.42%	1.86%	1.82%	2.28%
Fatty Acid Derivatives (N03AG)	1.44%	1.44%	1.53%	1.58%	1.42%
Benzodiazepine Derivatives (N03AE)	1.32%	1.28%	1.37%	1.28%	1.53%
Anticholinergics (R03BB)	1.31%	1.21%	1.48%	1.38%	1.86%
Vitamin B1, Plain (A11DA)	1.29%	1.17%	1.75%	1.41%	1.94%
Non-Selective Monoamine Reuptake Inhibitors (N06AA)	1.27%	1.24%	1.34%	1.21%	1.46%
Diazepines, Oxazepines, Thiazepines And Oxepines (N05AH)	1.23%	1.18%	1.41%	1.40%	1.54%
Angiotensin II Receptor Blockers (ARBs) And Diuretics (C09DA)	1.23%	1.15%	1.47%	1.49%	1.67%
Aldosterone Antagonists (C03DA)	1.23%	1.06%	1.70%	1.47%	2.10%
Selective Beta-2-Adrenoreceptor Agonists (R03AC)	1.19%	1.12%	1.37%	1.34%	1.54%
Penicillins With Extended Spectrum (J01CA)	1.19%	1.20%	1.17%	1.14%	1.13%
Xanthines (R03DA)	1.16%	1.08%	1.33%	1.44%	1.56%
Preparations Inhibiting Uric Acid Production (M04AA)	1.15%	0.95%	1.67%	1.38%	2.16%
Beta Blocking Agents, Non-Selective (C07AA)	1.12%	1.11%	1.32%	1.20%	1.17%
Thiazides, Plain (C03AA)	1.05%	0.98%	1.17%	1.08%	1.43%
Macrolides (J01FA)	1.01%	1.00%	1.00%	0.94%	1.05%

Supplementary Figure 1. Percentage of medication groups use totally and by outcomes in hospitalized COVID-19 patients.
