# Supplementary material for: Effect of the chronic medication use on outcome measures of hospitalized COVID-19 patients: Evidence from big data
Source: Front Public Health. 2023 Feb 24;11:1061307. doi: 10.3389/fpubh.2023.1061307 (PMC9998941; doi:10.3389/fpubh.2023.1061307)
Supplement: Supplementary file 6 [file Data_Sheet_2.PDF]

Medication Name (ATC)	Total	Recovered	ICU Admission	Ventilation Therapy	Death
Aspirin (B01AC06)	10.75%	9.88%	12.60%	12.55%	15.31%
Atorvastatin (C10AA05)	9.25%	8.62%	11.01%	10.88%	12.52%
Losartan (C09CA01)	8.01%	7.41%	9.64%	9.36%	11.09%
Metformin (A10BA02)	6.34%	6.08%	6.97%	6.89%	7.69%
Metoprolol (C07AB02)	4.85%	4.52%	5.80%	5.65%	6.58%
Glyceryl Trinitrate (C01DA02)	4.80%	4.28%	6.24%	5.56%	7.51%
Amlodipine (C08CA01)	4.58%	4.18%	5.52%	5.50%	6.65%
Furosemide (C03CA01)	3.45%	2.93%	4.87%	4.51%	6.18%
Ranitidine (A02BA02)	2.94%	2.75%	3.45%	3.46%	3.92%
Omeprazole (A02BC01)	2.93%	2.77%	3.20%	3.22%	3.76%
Insulin Glargine (A10AE04)	2.58%	2.36%	3.08%	2.88%	3.75%
Prednisolone (H02AB06)	2.33%	2.15%	2.29%	2.31%	3.27%
Carvedilol (C07AG02)	2.15%	1.93%	2.88%	2.60%	3.29%
Insulin Aspart (A10AB05)	2.01%	1.83%	2.45%	2.11%	2.90%
Glibenclamide (A10BB01)	1.98%	1.86%	2.27%	2.37%	2.64%
Levothyroxine Sodium (H03AA01)	1.92%	1.88%	2.22%	2.05%	2.13%
Gabapentin (N03AX12)	1.87%	1.75%	2.11%	2.23%	2.55%
Diclofenac (M01AB05)	1.73%	1.64%	1.69%	1.82%	2.16%
Piroxicam (M01AC01)	1.71%	1.68%	1.75%	1.65%	1.85%
Cefixime (J01DD08)	1.70%	1.65%	1.83%	1.81%	1.97%
Valsartan (C09CA03)	1.64%	1.53%	1.99%	1.66%	2.24%
Colecalciferol (A11CC05)	1.63%	1.56%	1.59%	1.74%	1.96%
Insulin Aspart (A10AD05)	1.62%	1.47%	2.07%	1.97%	2.42%
Folic Acid (B03BB01)	1.62%	1.48%	1.78%	1.79%	2.34%
Alprazolam (N05BA12)	1.60%	1.51%	1.85%	1.74%	2.08%
Valproic Acid (N03AG01)	1.44%	1.44%	1.53%	1.58%	1.42%
Salmeterol And Fluticasone (R03AK06)	1.42%	1.33%	1.56%	1.48%	1.92%
Clonazepam (N03AE01)	1.32%	1.28%	1.37%	1.28%	1.53%
Thiamine (A11DA01)	1.29%	1.17%	1.75%	1.41%	1.94%
Losartan And Diuretics (C09DA01)	1.23%	1.15%	1.47%	1.49%	1.67%
Spironolactone (C03DA01)	1.23%	1.06%	1.70%	1.47%	2.10%
Amoxicillin (J01CA04)	1.19%	1.20%	1.17%	1.14%	1.13%
Dexamethasone (H02AB02)	1.18%	1.07%	1.53%	1.41%	1.77%
Captopril (C09AA01)	1.17%	1.06%	1.37%	1.40%	1.73%
Allopurinol (M04AA01)	1.15%	0.95%	1.67%	1.38%	2.16%
Gliclazide (A10BB09)	1.12%	1.07%	1.28%	1.25%	1.40%
Propranolol (C07AA05)	1.10%	1.09%	1.29%	1.17%	1.16%
Calcitriol (A11CC04)	1.10%	0.93%	1.40%	1.22%	1.98%
Salbutamol (R03AC02)	1.07%	1.02%	1.24%	1.26%	1.37%
Hydrochlorothiazide (C03AA03)	1.05%	0.98%	1.17%	1.08%	1.43%
Betamethasone (H02AB01)	1.01%	1.02%	1.06%	1.07%	0.97%
Azithromycin (J01FA10)	1.01%	1.00%	0.99%	0.94%	1.05%

Supplementary Figure 2. Percentage of medications use totally and by outcomes in hospitalized COVID-19 patients.
