# Supplementary material for: Effect of the chronic medication use on outcome measures of hospitalized COVID-19 patients: Evidence from big data
Source: Front Public Health. 2023 Feb 24;11:1061307. doi: 10.3389/fpubh.2023.1061307 (PMC9998941; doi:10.3389/fpubh.2023.1061307)
Supplement: Supplementary file 7 [file Data_Sheet_3.PDF]

Set Length	Set Medications	Total	Recovered	ICU Admission	Ventilation Therapy	Death
Two-Medication Sets	Aspirin-Atorvastatin	5.75%	5.30%	6.77%	6.96%	8.08%
	Aspirin-Losartan	3.97%	3.63%	4.67%	4.72%	5.75%
	Atorvastatin-Losartan	3.62%	3.34%	4.33%	4.28%	5.09%
	Aspirin-Glyceryl Trinitrate	3.34%	3.00%	4.13%	3.83%	5.09%
	Atorvastatin-Metformin	3.05%	2.92%	3.48%	3.65%	3.71%
	Aspirin-Metoprolol	2.88%	2.67%	3.41%	3.58%	3.95%
	Aspirin-Metformin	2.81%	2.65%	3.18%	3.29%	3.63%
	Atorvastatin-Glyceryl Trinitrate	2.72%	2.46%	3.64%	3.30%	4.11%
	Atorvastatin-Metoprolol	2.48%	2.31%	3.01%	3.07%	3.37%
	Amlodipine-Aspirin	2.27%	2.06%	2.81%	2.78%	3.34%
	Losartan-Metformin	2.26%	2.15%	2.64%	2.64%	2.86%
	Amlodipine-Atorvastatin	2.01%	1.85%	2.55%	2.48%	2.81%
	Losartan-Metoprolol	2.00%	1.87%	2.45%	2.38%	2.71%
	Glyceryl Trinitrate-Losartan	1.97%	1.75%	2.57%	2.25%	3.12%
	Aspirin-Furosemide	1.92%	1.63%	2.59%	2.50%	3.44%
	Amlodipine-Losartan	1.78%	1.63%	2.09%	2.11%	2.58%
	Insulin Aspart-Insulin Glargine	1.62%	1.48%	1.97%	1.73%	2.38%
	Atorvastatin-Furosemide	1.57%	1.34%	2.09%	2.06%	2.78%
	Glyceryl Trinitrate-Metoprolol	1.56%	1.43%	1.98%	1.94%	2.25%
	Aspirin-Carvedilol	1.45%	1.31%	1.92%	1.73%	2.22%
	Glibenclamide-Metformin	1.44%	1.36%	1.58%	1.65%	1.84%
	Aspirin-Insulin Aspart	1.44%	1.25%	1.85%	1.76%	2.42%
	Atorvastatin-Insulin Aspart	1.42%	1.26%	1.82%	1.70%	2.30%
	Metformin-Metoprolol	1.28%	1.22%	1.47%	1.43%	1.58%
	Furosemide-Losartan	1.26%	1.06%	1.83%	1.70%	2.27%
	Amlodipine-Metoprolol	1.23%	1.13%	1.55%	1.51%	1.80%
	Atorvastatin-Carvedilol	1.20%	1.08%	1.65%	1.45%	1.82%
	Furosemide-Glyceryl Trinitrate	1.17%	0.98%	1.66%	1.56%	2.20%
	Amlodipine-Metformin	1.10%	1.03%	1.29%	1.33%	1.45%
	Amlodipine-Glyceryl Trinitrate	1.09%	0.96%	1.52%	1.36%	1.77%
	Glyceryl Trinitrate-Metformin	1.06%	0.98%	1.34%	1.26%	1.52%
	Insulin Aspart-Losartan	1.04%	0.93%	1.28%	1.24%	1.60%
	Atorvastatin-Insulin Glargine	1.03%	0.92%	1.24%	1.22%	1.64%
	Aspirin-Insulin Glargine	1.03%	0.90%	1.30%	1.24%	1.73%
	Insulin Aspart-Metformin	0.97%	0.90%	1.06%	1.13%	1.31%
	Carvedilol-Glyceryl Trinitrate	0.92%	0.80%	1.32%	1.14%	1.55%
	Atorvastatin-Glibenclamide	0.92%	0.86%	0.97%	1.11%	1.19%
	Carvedilol-Furosemide	0.90%	0.77%	1.32%	1.16%	1.54%
	Aspirin-Glibenclamide	0.89%	0.82%	1.04%	1.11%	1.21%
	Aspirin-Valsartan	0.88%	0.81%	1.10%	0.90%	1.24%
	Carvedilol-Losartan	0.84%	0.76%	1.14%	1.00%	1.28%
	Amlodipine-Furosemide	0.82%	0.70%	1.12%	1.20%	1.46%
	Atorvastatin-Valsartan	0.80%	0.73%	1.00%	0.88%	1.13%
	Furosemide-Spironolactone	0.77%	0.66%	1.08%	0.96%	1.36%
	Aspirin-Gabapentin	0.77%	0.68%	0.92%	0.99%	1.20%
	Gliclazide-Metformin	0.76%	0.73%	0.85%	0.82%	0.89%
	Insulin Glargine-Losartan	0.76%	0.68%	0.81%	0.86%	1.17%
	Atorvastatin-Levothyroxine Sodium	0.75%	0.71%	0.99%	0.87%	0.93%
	Aspirin-Spironolactone	0.74%	0.65%	0.93%	0.87%	1.22%
	Atorvastatin-Gabapentin	0.73%	0.66%	0.88%	0.92%	1.09%
	Insulin Glargine-Metformin	0.72%	0.68%	0.78%	0.88%	0.93%
	Glibenclamide-Losartan	0.72%	0.67%	0.81%	0.78%	0.98%
	Aspirin-Levothyroxine Sodium	0.71%	0.68%	0.87%	0.79%	0.90%
	Alprazolam-Aspirin	0.68%	0.62%	0.79%	0.75%	0.95%
	Furosemide-Metoprolol	0.65%	0.55%	0.87%	0.92%	1.17%
	Aspirin-Losartan And Diuretics	0.64%	0.59%	0.80%	0.77%	0.90%
	Aspirin-Captopril	0.62%	0.56%	0.68%	0.78%	0.92%
	Amlodipine-Valsartan	0.62%	0.58%	0.75%	0.64%	0.83%
	Alprazolam-Atorvastatin	0.61%	0.57%	0.71%	0.66%	0.82%
	Atorvastatin-Gliclazide	0.59%	0.57%	0.69%	0.76%	0.72%
	Atorvastatin-Losartan And Diuretics	0.58%	0.55%	0.66%	0.71%	0.78%
	Aspirin-Hydrochlorothiazide	0.58%	0.53%	0.70%	0.63%	0.87%
	Levothyroxine Sodium-Losartan	0.58%	0.56%	0.75%	0.68%	0.68%
	Aspirin-Diclofenac	0.57%	0.52%	0.59%	0.66%	0.83%
	Aspirin-Thiamine (Vit B1)	0.57%	0.50%	0.81%	0.62%	0.92%

Two-Medication Sets	Allopurinol-Aspirin	0.56%	0.45%	0.81%	0.66%	1.15%
	Allopurinol-Atorvastatin	0.56%	0.46%	0.84%	0.75%	1.12%
	Atorvastatin-Spironolactone	0.56%	0.49%	0.78%	0.73%	0.94%
	Folic Acid-Prednisolone	0.55%	0.52%	0.50%	0.57%	0.74%
	Amlodipine-Carvedilol	0.55%	0.49%	0.71%	0.69%	0.88%
	Aspirin-Folic Acid	0.55%	0.48%	0.72%	0.69%	0.88%
	Atorvastatin-Folic Acid	0.54%	0.48%	0.70%	0.68%	0.86%
	Atorvastatin-Thiamine (Vit B1)	0.53%	0.48%	0.71%	0.60%	0.82%
	Gabapentin-Losartan	0.53%	0.49%	0.69%	0.66%	0.73%
	Atorvastatin-Diclofenac	0.53%	0.49%	0.55%	0.65%	0.74%
	Aspirin-Gliclazide	0.52%	0.49%	0.63%	0.63%	0.68%
	Alprazolam-Losartan	0.52%	0.47%	0.59%	0.60%	0.76%
	Levothyroxine Sodium-Metformin	0.52%	0.50%	0.65%	0.54%	0.59%
	Atorvastatin-Captopril	0.52%	0.47%	0.66%	0.61%	0.76%
	Hydrochlorothiazide-Losartan	0.51%	0.47%	0.58%	0.55%	0.75%
	Atorvastatin-Prednisolone	0.51%	0.44%	0.55%	0.52%	0.84%
	Atorvastatin-Hydrochlorothiazide	0.51%	0.47%	0.59%	0.54%	0.69%
	Furosemide-Insulin Glargine	0.49%	0.40%	0.68%	0.62%	0.99%
	Diclofenac-Losartan	0.49%	0.45%	0.49%	0.54%	0.68%
	Aspirin-Colecalciferol	0.48%	0.44%	0.52%	0.58%	0.68%
	Atorvastatin-Colecalciferol	0.48%	0.45%	0.50%	0.54%	0.63%
	Glyceryl Trinitrate-Insulin Glargine	0.48%	0.40%	0.63%	0.58%	0.86%
	Furosemide-Metformin	0.47%	0.41%	0.62%	0.58%	0.80%
	Allopurinol-Furosemide	0.47%	0.36%	0.73%	0.65%	1.02%
	Losartan-Prednisolone	0.47%	0.42%	0.50%	0.47%	0.73%
	Aspirin-Prednisolone	0.46%	0.40%	0.53%	0.51%	0.79%
	Aspirin-Cefixime	0.46%	0.43%	0.55%	0.46%	0.64%
	Glyceryl Trinitrate-Spironolactone	0.45%	0.38%	0.67%	0.62%	0.82%
	Amlodipine-Insulin Glargine	0.45%	0.40%	0.59%	0.61%	0.72%
	Metformin-Valsartan	0.45%	0.43%	0.48%	0.47%	0.56%
	Aspirin-Calcitriol	0.45%	0.35%	0.59%	0.51%	0.94%
	Metoprolol-Valsartan	0.45%	0.41%	0.49%	0.46%	0.62%
	Colecalciferol-Losartan	0.44%	0.41%	0.41%	0.44%	0.61%
	Aspirin-Piroxicam	0.44%	0.41%	0.47%	0.42%	0.60%
	Carvedilol-Spironolactone	0.44%	0.39%	0.61%	0.51%	0.68%
	Atorvastatin-Calcitriol	0.44%	0.36%	0.60%	0.49%	0.84%
	Diclofenac-Piroxicam	0.43%	0.41%	0.41%	0.38%	0.54%
	Folic Acid-Losartan	0.43%	0.39%	0.59%	0.51%	0.64%
	Carvedilol-Metformin	0.42%	0.39%	0.53%	0.47%	0.60%
	Gliclazide-Losartan	0.42%	0.39%	0.53%	0.61%	0.59%
	Glyceryl Trinitrate-Valsartan	0.42%	0.38%	0.47%	0.38%	0.66%
	Gabapentin-Metformin	0.42%	0.40%	0.44%	0.50%	0.54%
	Atorvastatin-Cefixime	0.42%	0.39%	0.49%	0.48%	0.60%
	Calcitriol-Furosemide	0.42%	0.33%	0.61%	0.54%	0.87%
	Allopurinol-Losartan	0.41%	0.34%	0.60%	0.50%	0.78%
	Insulin Glargine-Metoprolol	0.41%	0.36%	0.44%	0.50%	0.64%
	Losartan-Thiamine (Vit B1)	0.41%	0.37%	0.55%	0.53%	0.61%
	Aspirin-Salmeterol And Fluticasone	0.40%	0.36%	0.44%	0.40%	0.61%
	Losartan-Spironolactone	0.40%	0.34%	0.63%	0.54%	0.67%
	Glibenclamide-Metoprolol	0.39%	0.37%	0.47%	0.51%	0.51%
	Aspirin-Clonazepam	0.39%	0.35%	0.46%	0.38%	0.62%
	Losartan And Diuretics-Metformin	0.39%	0.37%	0.40%	0.43%	0.51%
	Losartan-Piroxicam	0.39%	0.37%	0.40%	0.41%	0.52%
	Furosemide-Insulin Aspart	0.39%	0.31%	0.55%	0.48%	0.81%
	Cefixime-Losartan	0.39%	0.36%	0.49%	0.44%	0.52%
	Atorvastatin-Piroxicam	0.38%	0.36%	0.37%	0.34%	0.45%
	Furosemide-Valsartan	0.38%	0.33%	0.54%	0.45%	0.63%
	Metformin-Thiamine (Vit B1)	0.37%	0.34%	0.48%	0.37%	0.55%
	Amlodipine-Calcitriol	0.37%	0.32%	0.51%	0.43%	0.65%
	Losartan And Diuretics-Metoprolol	0.37%	0.34%	0.46%	0.46%	0.50%
	Amlodipine-Glibenclamide	0.37%	0.33%	0.43%	0.47%	0.56%
	Alprazolam-Glyceryl Trinitrate	0.37%	0.33%	0.53%	0.46%	0.57%
	Diclofenac-Metformin	0.37%	0.34%	0.29%	0.40%	0.48%
	Amlodipine-Losartan And Diuretics	0.36%	0.34%	0.48%	0.48%	0.49%
	Colecalciferol-Piroxicam	0.36%	0.35%	0.37%	0.41%	0.40%
	Levothyroxine Sodium-Metoprolol	0.36%	0.34%	0.39%	0.43%	0.46%
	Glibenclamide-Glyceryl Trinitrate	0.36%	0.32%	0.44%	0.43%	0.57%

Two-Medication Sets	Glyceryl Trinitrate-Insulin Aspart	0.36%	0.30%	0.50%	0.38%	0.68%
	Folic Acid-Furosemide	0.36%	0.30%	0.48%	0.49%	0.66%
	Atorvastatin-Clonazepam	0.35%	0.32%	0.41%	0.34%	0.52%
	Calcitriol-Losartan	0.35%	0.30%	0.49%	0.37%	0.62%
	Alprazolam-Metoprolol	0.35%	0.32%	0.43%	0.41%	0.45%
	Amoxicillin-Aspirin	0.34%	0.33%	0.33%	0.35%	0.40%
	Salbutamol-Salmeterol And Fluticasone	0.34%	0.32%	0.30%	0.34%	0.46%
	Gabapentin-Glyceryl Trinitrate	0.34%	0.30%	0.43%	0.40%	0.57%
	Amlodipine-Hydrochlorothiazide	0.34%	0.32%	0.32%	0.33%	0.43%
	Betamethasone-Piroxicam	0.34%	0.33%	0.32%	0.36%	0.37%
	Gabapentin-Metoprolol	0.33%	0.31%	0.42%	0.51%	0.46%
	Losartan-Salmeterol And Fluticasone	0.33%	0.30%	0.39%	0.34%	0.52%
	Amlodipine-Insulin Aspart	0.33%	0.30%	0.45%	0.39%	0.51%
	Aspirin-Valproic Acid	0.33%	0.31%	0.41%	0.34%	0.43%
	Alprazolam-Metformin	0.33%	0.31%	0.29%	0.31%	0.43%
	Hydrochlorothiazide-Metoprolol	0.33%	0.31%	0.38%	0.33%	0.41%
	Allopurinol-Amlodipine	0.32%	0.28%	0.49%	0.40%	0.56%
	Atorvastatin-Salmeterol And Fluticasone	0.32%	0.29%	0.39%	0.34%	0.52%
	Hydrochlorothiazide-Metformin	0.32%	0.31%	0.27%	0.33%	0.40%
	Amoxicillin-Atorvastatin	0.32%	0.32%	0.32%	0.29%	0.33%
	Amlodipine-Folic Acid	0.32%	0.29%	0.35%	0.39%	0.50%
	Captopril-Metformin	0.32%	0.29%	0.39%	0.44%	0.48%
	Amlodipine-Levothyroxine Sodium	0.32%	0.30%	0.38%	0.34%	0.41%
	Amlodipine-Gabapentin	0.32%	0.29%	0.43%	0.53%	0.46%
Three-Medication Sets	Aspirin-Atorvastatin-Losartan	2.45%	2.25%	2.83%	2.95%	3.50%
	Aspirin-Atorvastatin-Glyceryl Trinitrate	2.17%	1.97%	2.78%	2.63%	3.20%
	Aspirin-Atorvastatin-Metformin	1.87%	1.78%	2.17%	2.38%	2.38%
	Aspirin-Atorvastatin-Metoprolol	1.85%	1.72%	2.20%	2.35%	2.52%
	Aspirin-Glyceryl Trinitrate-Losartan	1.45%	1.30%	1.81%	1.68%	2.19%
	Amlodipine-Aspirin-Atorvastatin	1.37%	1.26%	1.76%	1.73%	1.92%
	Atorvastatin-Losartan-Metformin	1.35%	1.28%	1.64%	1.72%	1.72%
	Aspirin-Losartan-Metformin	1.27%	1.20%	1.49%	1.62%	1.64%
	Aspirin-Losartan-Metoprolol	1.27%	1.17%	1.57%	1.61%	1.76%
	Atorvastatin-Glyceryl Trinitrate-Losartan	1.23%	1.11%	1.60%	1.47%	1.88%
	Aspirin-Glyceryl Trinitrate-Metoprolol	1.19%	1.11%	1.45%	1.49%	1.64%
	Aspirin-Atorvastatin-Furosemide	1.16%	1.00%	1.49%	1.49%	2.03%
	Atorvastatin-Losartan-Metoprolol	1.13%	1.05%	1.41%	1.42%	1.54%
	Atorvastatin-Glyceryl Trinitrate-Metoprolol	1.03%	0.96%	1.25%	1.33%	1.41%
	Aspirin-Atorvastatin-Insulin Aspart	0.97%	0.84%	1.29%	1.21%	1.64%
	Amlodipine-Aspirin-Losartan	0.97%	0.88%	1.10%	1.20%	1.41%
	Aspirin-Atorvastatin-Carvedilol	0.95%	0.86%	1.28%	1.15%	1.43%
	Amlodipine-Atorvastatin-Losartan	0.88%	0.81%	1.01%	1.08%	1.24%
	Aspirin-Furosemide-Glyceryl Trinitrate	0.87%	0.74%	1.17%	1.13%	1.55%
	Aspirin-Metformin-Metoprolol	0.83%	0.80%	0.95%	1.06%	1.04%
	Atorvastatin-Metformin-Metoprolol	0.82%	0.79%	0.93%	1.02%	1.02%
	Amlodipine-Aspirin-Glyceryl Trinitrate	0.81%	0.72%	1.10%	0.99%	1.27%
	Aspirin-Glyceryl Trinitrate-Metformin	0.81%	0.75%	1.03%	1.03%	1.10%
	Aspirin-Furosemide-Losartan	0.80%	0.67%	1.06%	1.02%	1.44%
	Amlodipine-Aspirin-Metoprolol	0.76%	0.69%	0.98%	1.01%	1.09%
	Atorvastatin-Glyceryl Trinitrate-Metformin	0.73%	0.68%	0.91%	0.98%	0.98%
	Aspirin-Carvedilol-Glyceryl Trinitrate	0.72%	0.63%	1.03%	0.88%	1.19%
	Atorvastatin-Furosemide-Glyceryl Trinitrate	0.72%	0.61%	1.02%	0.96%	1.28%
	Glyceryl Trinitrate-Losartan-Metoprolol	0.72%	0.65%	0.92%	0.87%	1.06%
	Atorvastatin-Furosemide-Losartan	0.70%	0.60%	0.89%	0.86%	1.26%
	Atorvastatin-Glibenclamide-Metformin	0.70%	0.67%	0.76%	0.83%	0.86%
	Aspirin-Atorvastatin-Insulin Glargine	0.70%	0.60%	0.89%	0.86%	1.19%
	Amlodipine-Atorvastatin-Glyceryl Trinitrate	0.69%	0.62%	0.98%	0.89%	1.02%
	Aspirin-Glibenclamide-Metformin	0.67%	0.63%	0.76%	0.79%	0.89%
	Amlodipine-Aspirin-Metformin	0.66%	0.62%	0.76%	0.81%	0.83%
	Aspirin-Insulin Aspart-Insulin Glargine	0.65%	0.55%	0.85%	0.75%	1.17%
	Amlodipine-Atorvastatin-Metoprolol	0.65%	0.61%	0.88%	0.87%	0.90%
	Atorvastatin-Insulin Aspart-Insulin Glargine	0.65%	0.56%	0.78%	0.71%	1.10%
	Amlodipine-Atorvastatin-Metformin	0.65%	0.62%	0.80%	0.86%	0.76%
	Aspirin-Carvedilol-Furosemide	0.64%	0.55%	0.91%	0.84%	1.10%
	Losartan-Metformin-Metoprolol	0.62%	0.60%	0.76%	0.78%	0.74%
	Atorvastatin-Carvedilol-Glyceryl Trinitrate	0.60%	0.53%	0.91%	0.76%	0.99%
	Aspirin-Carvedilol-Losartan	0.60%	0.54%	0.80%	0.73%	0.90%

Three-Medication Sets	Aspirin-Atorvastatin-Valsartan	0.56%	0.51%	0.72%	0.61%	0.82%
	Aspirin-Atorvastatin-Glibenclamide	0.56%	0.52%	0.61%	0.74%	0.75%
	Amlodipine-Losartan-Metoprolol	0.54%	0.49%	0.68%	0.71%	0.81%
	Glibenclamide-Losartan-Metformin	0.54%	0.51%	0.63%	0.61%	0.69%
	Furosemide-Glyceryl Trinitrate-Losartan	0.54%	0.46%	0.74%	0.71%	0.98%
	Atorvastatin-Carvedilol-Furosemide	0.53%	0.46%	0.77%	0.71%	0.91%
	Glyceryl Trinitrate-Losartan-Metformin	0.52%	0.48%	0.70%	0.65%	0.76%
	Amlodipine-Losartan-Metformin	0.52%	0.49%	0.59%	0.66%	0.65%
	Atorvastatin-Carvedilol-Losartan	0.52%	0.46%	0.68%	0.62%	0.79%
	Aspirin-Atorvastatin-Gabapentin	0.49%	0.44%	0.60%	0.67%	0.79%
	Amlodipine-Glyceryl Trinitrate-Losartan	0.49%	0.43%	0.62%	0.63%	0.83%
	Insulin Aspart-Insulin Glargine-Losartan	0.49%	0.43%	0.54%	0.55%	0.78%
	Aspirin-Furosemide-Spironolactone	0.49%	0.42%	0.64%	0.58%	0.82%
	Amlodipine-Aspirin-Furosemide	0.48%	0.40%	0.70%	0.71%	0.88%
	Atorvastatin-Insulin Glargine-Losartan	0.47%	0.41%	0.50%	0.58%	0.74%
	Aspirin-Insulin Glargine-Losartan	0.46%	0.40%	0.51%	0.57%	0.76%
	Aspirin-Atorvastatin-Spironolactone	0.44%	0.39%	0.58%	0.59%	0.73%
	Aspirin-Atorvastatin-Levothyroxine Sodium	0.44%	0.42%	0.54%	0.51%	0.57%
	Carvedilol-Furosemide-Glyceryl Trinitrate	0.43%	0.37%	0.68%	0.62%	0.79%
	Amlodipine-Atorvastatin-Furosemide	0.43%	0.37%	0.59%	0.67%	0.77%
	Atorvastatin-Insulin Glargine-Metformin	0.43%	0.41%	0.48%	0.51%	0.55%
	Atorvastatin-Gliclazide-Metformin	0.43%	0.42%	0.51%	0.53%	0.49%
	Glyceryl Trinitrate-Metformin-Metoprolol	0.43%	0.40%	0.51%	0.54%	0.57%
	Amlodipine-Glyceryl Trinitrate-Metoprolol	0.42%	0.37%	0.54%	0.55%	0.65%
	Carvedilol-Glyceryl Trinitrate-Losartan	0.41%	0.36%	0.58%	0.48%	0.71%
	Aspirin-Furosemide-Metoprolol	0.41%	0.35%	0.55%	0.61%	0.74%
	Alprazolam-Aspirin-Atorvastatin	0.41%	0.38%	0.51%	0.53%	0.58%
	Insulin Aspart-Insulin Glargine-Metformin	0.41%	0.38%	0.46%	0.51%	0.55%
	Atorvastatin-Glibenclamide-Losartan	0.40%	0.38%	0.44%	0.44%	0.53%
	Aspirin-Atorvastatin-Losartan And Diuretics	0.40%	0.37%	0.50%	0.51%	0.55%
	Aspirin-Glibenclamide-Losartan	0.40%	0.38%	0.46%	0.48%	0.52%
	Atorvastatin-Furosemide-Metoprolol	0.39%	0.33%	0.52%	0.58%	0.69%
	Aspirin-Insulin Glargine-Metformin	0.38%	0.36%	0.44%	0.52%	0.53%
	Aspirin-Gliclazide-Metformin	0.38%	0.36%	0.43%	0.45%	0.45%
	Aspirin-Atorvastatin-Captopril	0.38%	0.34%	0.45%	0.48%	0.56%
	Amlodipine-Aspirin-Carvedilol	0.37%	0.33%	0.50%	0.48%	0.61%
	Carvedilol-Furosemide-Losartan	0.37%	0.32%	0.55%	0.45%	0.63%
	Aspirin-Atorvastatin-Gliclazide	0.37%	0.35%	0.44%	0.51%	0.47%
	Aspirin-Atorvastatin-Hydrochlorothiazide	0.37%	0.33%	0.45%	0.41%	0.53%
	Allopurinol-Aspirin-Atorvastatin	0.36%	0.29%	0.51%	0.44%	0.73%
	Aspirin-Atorvastatin-Thiamine (Vit B1)	0.36%	0.32%	0.47%	0.38%	0.55%
	Amlodipine-Aspirin-Valsartan	0.36%	0.33%	0.42%	0.36%	0.48%
	Atorvastatin-Furosemide-Spironolactone	0.36%	0.31%	0.48%	0.45%	0.60%
	Amlodipine-Furosemide-Losartan	0.36%	0.31%	0.45%	0.51%	0.61%
	Aspirin-Glyceryl Trinitrate-Insulin Glargine	0.35%	0.30%	0.45%	0.41%	0.63%
	Aspirin-Insulin Aspart-Losartan	0.35%	0.30%	0.41%	0.43%	0.62%
	Atorvastatin-Insulin Aspart-Losartan	0.35%	0.31%	0.41%	0.42%	0.59%
	Amlodipine-Metformin-Metoprolol	0.35%	0.34%	0.41%	0.41%	0.42%
	Aspirin-Glyceryl Trinitrate-Spironolactone	0.35%	0.30%	0.47%	0.49%	0.61%
	Aspirin-Atorvastatin-Folic Acid	0.33%	0.30%	0.46%	0.45%	0.52%
	Aspirin-Carvedilol-Spironolactone	0.33%	0.29%	0.43%	0.38%	0.51%
	Aspirin-Furosemide-Metformin	0.33%	0.28%	0.45%	0.45%	0.56%
	Atorvastatin-Glyceryl Trinitrate-Insulin Glargine	0.33%	0.27%	0.47%	0.43%	0.61%
	Amlodipine-Atorvastatin-Carvedilol	0.33%	0.29%	0.44%	0.41%	0.52%
	Furosemide-Insulin Aspart-Insulin Glargine	0.32%	0.25%	0.46%	0.39%	0.69%
	Aspirin-Gabapentin-Losartan	0.32%	0.30%	0.43%	0.41%	0.46%
	Aspirin-Furosemide-Insulin Glargine	0.32%	0.26%	0.44%	0.39%	0.66%
	Aspirin-Atorvastatin-Diclofenac	0.32%	0.29%	0.34%	0.40%	0.48%
	Atorvastatin-Levothyroxine Sodium-Losartan	0.32%	0.30%	0.43%	0.39%	0.41%
	Atorvastatin-Gabapentin-Losartan	0.32%	0.29%	0.45%	0.40%	0.45%
	Aspirin-Carvedilol-Metformin	0.32%	0.29%	0.44%	0.38%	0.45%
	Aspirin-Glyceryl Trinitrate-Valsartan	0.32%	0.28%	0.33%	0.28%	0.49%
	Amlodipine-Atorvastatin-Valsartan	0.32%	0.29%	0.41%	0.34%	0.44%

Supplementary Figure 3. Percentage of medication sets use totally and by outcomes in hospitalized COVID-19 patients.
